# Hepatoprotective Effect and Potential Mechanism of Aqueous Extract from *Phyllanthus emblica* on Carbon-Tetrachloride-Induced Liver Fibrosis in Rats

**DOI:** 10.1155/2021/5345821

**Published:** 2021-10-19

**Authors:** Kehuan Yin, Xuedong Li, Xiaomin Luo, Yuru Sha, Puyang Gong, Jian Gu, Rui Tan

**Affiliations:** ^1^College of Pharmacy, Southwest Minzu University, 610041 Chengdu, China; ^2^College of Life Science and Engineering, Southwest Jiaotong University, 610031 Chengdu, China

## Abstract

Liver fibrosis is a pathological variation caused by almost all chronic liver injuries. As an edible and medicinal natural resource, *Phyllanthus emblica* (PE) has been reported to possess hepatoprotective, antioxidant, and anti-inflammatory activities and may have an ameliorating effect on hepatic fibrosis. To investigate the protective effect of the aqueous extract of PE (AEPE) against liver fibrosis and to uncover its related mechanisms, the chemical profile of AEPE was characterized by high performance liquid chromatography (HPLC) and sulfuric acid-phenol method. Ameliorative effects of different doses of AEPE were investigated in carbon-tetrachloride- (CCl_4_-) induced liver fibrosis rats by analyzing biochemical markers, morphologic pathology, and related proteins expression in liver tissue. The results indicated that AEPE (1.8, 3.6 g/kg) could significantly reduce levels of alanine aminotransferase (ALT), aspartate aminotransferase (AST), collagen IV (Col IV), type III precollagen (PCIII), hyaluronic acid (HA), laminin (LN), malondialdehyde (MDA), nitric oxide (NO), protein carbonyl (PC), tumor necrosis factor-*α*(TNF-*α*), interleukin-1*β* (IL-1*β*), interleukin-6 (IL-6), and hydroxyproline (Hyp) and increase the levels of superoxide dismutase (SOD), glutathione (GSH), and catalase (CAT). Hematoxylin-eosin (H&E), Sirius red, and Masson staining showed AEPE-treated improved fibrotic lesions and inflammatory cell infiltration. Meanwhile, AEPE treatment also significantly downregulates the expression of *α*-smooth muscle actin (*α*-SMA) and transforming growth factor-*β*1 (TGF-*β*1) in the liver tissue and serum, respectively. In conclusion, AEPE possesses curative efficacy against liver fibrosis through its antioxidant, anti-inflammatory, and antifibrotic effects.

## 1. Introduction

Liver fibrosis (LF) is a pathophysiological process manifesting as amounts of liver parenchymal cells are replaced by fibrous connective tissue and may translate into cirrhosis even hepatocellular carcinoma [1, 2]. Chronic liver damage is caused by a variety of etiologies which further induce liver fibrosis, including alcohol toxicity, chemical poisons, chronic viruses, and immune damage [3]. Currently, clinical therapeutics of LF focus on either controlling the primary disease or treatment of liver fibrosis itself by routinely reducing inflammation, inhibiting oxidative stress, and promoting collagen degradation [4–7]. However, specific and high-efficiency antiliver fibrosis drugs with no obvious side effects have not yet appeared. In recent years, a number of medicinal plants have shown great advantages characterized by diverse bioactivities and low toxicity in the treatment of various types of liver diseases, which provides an important source for the development of antiliver fibrosis drugs [8, 9].

*Phyllanthus emblica* L. (PE) belongs to the family *Euphorbiaceae* which synonymously is named *Emblica officinalis*, also known as Indian gooseberry (Amla) and Yuganzi in India and China, respectively [10]. The dried production of PE has been applied as herbal medicine in traditional Chinese medicine, Indian medicine, and Tibetan medicine recorded in classic medical books for thousands of years [11, 12]. Modern phytochemical researches demonstrated that the main ingredients contained in PE are tannins, flavonoids, polyphenols, vitamins, amino acids, carbohydrates, proteins, and so on [10]. The diversity of chemical components leads to the diversity of its biological activity. PE extract possessed multiple biological activities including antimicrobial, antioxidant, anti-inflammatory, analgesic, antipyretic, and antitumor. [13, 14]. Notably, plenty of studies have shown that PE has a unique protective effect on liver injury induced by high fat diet and multiple hepatotoxic agents, such as ethanol, heavy metals, carbon tetrachloride, ochratoxins, antitubercular drugs, and hexachlorocyclohexane [1, 15–20]. Due to the outstanding hepatoprotective effect of PE, the prospect of its ability to inhibit liver fibrosis appears attractive.

Previous studies have preliminary shown that PE extract could reverse the early fibrotic pathologic changes in liver induced by CCl_4_ and thioacetamide (TAA) via its antioxidant property [21, 22]. The water extract of PE can significantly reduce the generation of reactive oxygen species (ROS) in HepG2 cells and inhibit the hepatic fibrosis of HSC-T6 cells induced by leptin [23]. However, the antiliver fibrosis effect of PE still needs to be confirmed *in vivo* by specific liver fibrosis biomarker detection and collagen pathological analysis. Meanwhile, chronic liver injury evolving into fibrosis also involved a variety of abnormal biological process, such as the massive release of inflammatory factors, oxidative stress, and excessive activation of hepatic stellate cells (HSC), as well as secretion and deposition of extracellular matrix (ECM) [2, 24, 25]. Hence, the complex mechanism of antihepatic fibrosis of PE still needs further exploration.

In the present study, 7 major representative compounds in AEPE were identified by HPLC analysis and its polysaccharide content was tested. Then, rats with liver fibrosis induced by carbon tetrachloride (CCl_4_) were used as model for exploring the effects of AEPE on hepatic fibrosis and its potential mechanism in vivo. This research aimed to provide data support for rationally exploiting and developing PE as a natural medicine for liver fibrosis disease treatment.

## 2. Materials and Methods

### 2.1. Chemicals and Reagents

The chemical reference standards, gallic acid, chlorogenic acid, methyl gallate, corilagin, chebulagic acid, ellagic acid, and chebulinic acid were provided by Sichuan Weikeqi Biological Technology Co., Ltd. (Chengdu, China). Formic acid (HPLC grade) was bought from Aladdin Industrial Corporation (Shanghai, China). Acetonitrile (HPLC grade) and olive oil (medicinal, ≥ 99.9%) were purchased from Sigma-Aldrich (St. Louis, MO, USA). Carbon tetrachloride (CCl_4_ ) (anhydrous, ≥ 99.0%) was bought from Shanghai Macklin Biochemical Co., Ltd. (Shanghai, China). Silymarin (Legalon®) was purchased from Madaus (Cologne, Germany). Commercial diagnostic kits of alanine aminotransferase (ALT), aspartate aminotransferase (AST), superoxide dismutase (SOD), malondialdehyde (MDA), nitric oxide (NO), catalase (CAT), and glutathione (GSH) and hydroxyproline (Hyp) assay kits were purchased from Nanjing Jiancheng Bioengineering Institute (Nanjing, China). TGF-*β*1 ELISA kits were purchased from MultiSciences Biotech Co., Ltd. (Hangzhou, China). Collagen IV (Col IV), type III precollagen (PC III), hyaluronic acid (HA), laminin (LN), protein carbonyl (PC), and TNF-*α*, TL-1*β*, and IL-6 ELISA kits were obtained by Shanghai Enzyme Link Biotechnology Co., Ltd. (Shanghai, China).

### 2.2. Preparation of AEPE

The dried and pitted fruit of PE was obtained from the Lotus Pond herbal medicine market in Sichuan province and was identified by Prof. Jian Gu at the College of Pharmacy, Southwest Minzu University. A voucher specimen (no. 20201212) was deposited at the herbarium of the College of Pharmacy, Southwest Minzu University.

The 1000 g PE dry powder was extracted with distilled water on a rotary shaker (150 rpm) for 24 h at 37°C. The extract was filtered, evaporated, and concentrated to obtain the AEPE at a concentration of 1 g (crude drug)/mL. The AEPE was further precipitated by adding 4 volumes of absolute ethyl alcohol for two times. The supernatant was combined and concentrated to obtain the small-molecule fraction (SMF) for HPLC analysis. The resulting precipitate was lyophilized and collected, and the crude polysaccharide was obtained. The total polysaccharide content of PE was tested by phenol-sulfuric acid method using glucose as the standard [26].

### 2.3. HPLC Analysis

The chromatographic separations of SMF and chemical reference standards were performed using a Waters T3 column (250 mm × 4.6 mm, 5 *μ*m) operated at 37 °C employing a gradient elution using 0.1% formic acid in water (*A*) and acetonitrile (B) as mobile phase at a flow rate of 0.6 mL/min. The optimized HPLC elution condition was as follows: isocratic 3% B (0–6 min), linear gradient from 3% to 4% B (6–15 min), 4%–14% B (15–20 min), isocratic 14% B (20–45 min), and linear gradient from 14% to 40% B (45–75 min). The detection wavelength was 254 nm, and injection volume was 5 *μ*L for all samples.

### 2.4. Animals Treatment

Male Wistar rats (200 ± 10 g) were supplied by Chengdu Dossy Experimental Animal Co., Ltd. (Sichuan, China). The animals were housed six per cage on cypress wood padding bedding in a barrier room with controlled temperature (22.0 ± 2°C), humidity (50–60%), free access to food and drinking water, an alternating 12-h room light and dark cycle. All animal procedures were approved by the Ethics Committee of Southwest Minzu University and were conducted in accordance with university guidelines.

After one week of acclimation, the animals were randomized into six groups (with 10 rats per group) including normal control group (Control), model group (Model), AEPE low-dose group (AEPE-L), AEPE middle-dose group (AEPE-M), AEPE high-dose group (AEPE-H), and silymarin group (Silymarin). The rats in the control group received 2 mL/kg olive oil twice a week for 8 weeks, and in the other 5 groups they were induced by intraperitoneally injecting CCl_4_-olive oil (1 : 1 v/v, 2 mL/kg) twice a week for 8 weeks [25]. Rats in the silymarin group were gavaged daily with silymarin dissolved in water at a dosage of 42 mg/kg. AEPE were dissolved in advance with distilled water and administered to the rats in AEPE-treated group by gavage (low, middle, and high at a dosage of 0.9 g/kg, 1.8 g/kg, and 3.6 g/kg of crude drug). Meanwhile, rats in the control and model group were daily given the same volume of distilled water through intragastric gavage.

After 8 weeks, all rats were anaesthetized with ether, blood was collected from the abdominal aorta, livers were collected and weighted, and the liver index was calculated and expressed as liver weight/final body weight. The blood samples were stored in -20°C after being centrifuged at 862*g* for 15 min. The left lobe of the liver was cut off and immersed in a tissue fixing solution (4% para formaldehyde solution) for subsequent staining and assessment. The rest of the liver tissue was stored at -80°C for further analysis.

### 2.5. Biochemical Analyses

Serum levels of ALT and AST were analyzed according to commercially available kit instructions.

### 2.6. Examination of Histopathology and Collagen Deposition of Liver Tissue

The liver tissues were fixed with 4% paraformaldehyde and were paraffin-embedded and sectioned. Hematoxylin-eosin (H&E) staining was used for the initial histopathological observation of hepatic injury and fibrosis. The criteria used to evaluate the severity of fibrosis are as follows: 0 points, normal; 1 point, presence of collagen fibers, extending from the portal triad or central vein to the peripheral area; 2 points, mild fibrosis, collagen fibers extending but no compartment formation; 3 points, moderate fibrosis, having some collagen fibers formed by pseudolobes; 4 points, severe fibrosis, increased collagen fibers, thickened parts of the compartment, and frequent pseudolobes formation [27].

Collagen deposition change in liver tissue was evaluated by Masson staining and Sirius red staining. Representative images were captured by an Olympus BX53 Microscope. The content of Hyp in liver tissue homogenates was measured according to the manufacturer's instructions.

### 2.7. Immunohistochemical Staining

The paraffin sections were deparaffinized; washed sequentially with xylene, alcohol, and distilled water; then used for antigen retrieval with citric acid antigen retrieval buffer; and then blocked with 3% bovine serum albumin (BSA) at room temperature for 30 minutes. Subsequently, the sections were incubated overnight at 4°C with the corresponding primary antibody. After washing 3 times with phosphate buffered solution (PBS), the liver sections were incubated with the secondary antibody for 50 minutes at room temperature. Then, the slices were washed 3 times with PBS, and freshly prepared diaminobenzidine (DAB) chromogenic solution was added, dehydrated, and fixed. Finally, the expression of *α*-SMA was observed and measured.

### 2.8. Oxidative Stress Parameters Detection

The activities of SOD and CAT and the contents of MDA, PC, GSH, and NO in rat serum and liver tissues were tested according to commercially available kit instructions.

### 2.9. Enzyme-Linked Immunosorbent Assay (ELISA) Analysis

Serum levels of hepatic fibrosis indexes, including HA, LN, IV-C, and PCIII, were measured by ELISA assay, as well as the expressions of cytokines of TGF-*β*1, TNF-*α*, IL-1*β,* and IL-6.

### 2.10. Statistical Analysis

Data were expressed as the means ± S.E.M. Differences between the groups were determined by one-way analysis of variance (ANOVA) followed by Dunnett's multiple comparisons as a post-hoc analysis test for comparison. Statistical analyses were performed with the GraphPad Prism 9.0 software, and *p*-value < 0.05 was deemed significant.

## 3. Results

### 3.1. Chemical Profile Characterizations of AEPE

**Figure 1:**
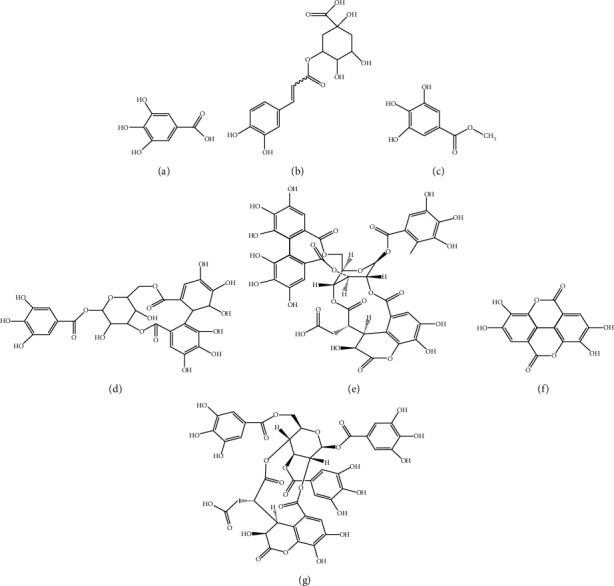
Structural formulas for qualitative detection of 7 chemical components in AEPE. (a) Gallic acid; (b) chlorogenic acid; (c) methyl gallate; (d) corilagin; (e) chebulagic acid; (f) ellagic acid; (g) chebulinic acid.

**Figure 2:**
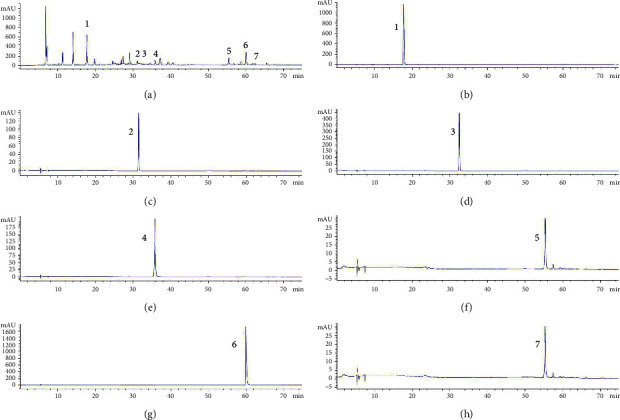
Representative high-performance liquid chromatograms of AEPE sample (a) and standard compounds (b–h). 1, Gallic acid; 2, chlorogenic acid; 3, methyl gallate; 4, corilagin; 5, chebulagic acid; 6, ellagic acid; and 7, chebulinic acid.

Seven chemical components were used as reference standards to qualitatively characterize AEPE by HPLC analysis, and the structures of them are shown in Figure 1. The presence of gallic acid, corilagin, methyl gallate, chlorogenic acid, chebulinic acid, chebulagic acid, and ellagic acid in AEPE was confirmed, and representative chromatograms are shown in Figure 2.

The content of polysaccharide in AEPE was detected by phenol-sulfuric acid method. Quantitative results illustrated that glucose content has a good linear relationship with the absorbance in the range of 0.00–0.20 mg/mL. The regression equation is *y* = 5.5202*x*+0.5935, *R*^2^ = 0.9994, and the polysaccharide content of AEPE is 3.13%.

### 3.2. AEPE Ameliorated the General Condition and Liver Pathological Changes

The rats in the control group had smooth coat color and active mental state, and their body mass increased regularly during the experiment. In comparison, the body hair of the rats in the model group is obviously yellow and lethargic, appetite decreased, and body weight is obviously reduced. The coat color, mental state, and food intake of rats treated by AEPE or silymarin were improved significantly.

**Figure 3:**
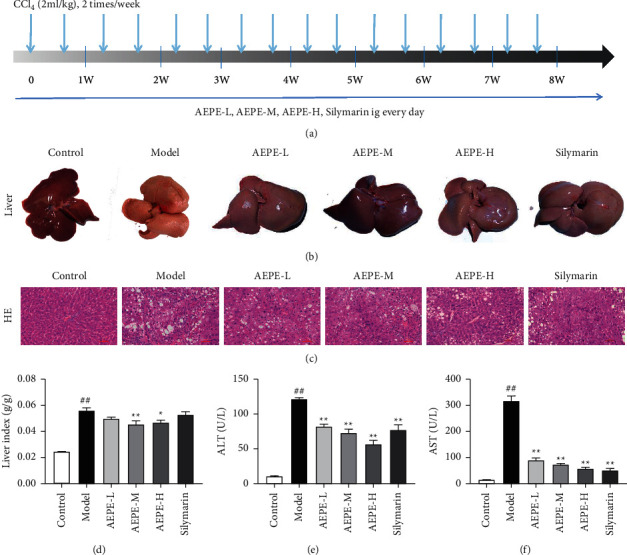
Effects of AEPE on liver appearance, histopathology, liver index, and liver function. (a) Graphic illustration of the experiment design. (b) Representative photograph of livers from each experiment group under macroscopic view. (c) Representative histology of liver tissue from each group by H&E staining under light microscopy at 200x magnifications. (d) Rats and their liver samples were weighted and liver-to-body weight ratios were calculated. (e) Effect of AEPE on serum ALT levels. (f) Effect of AEPE on serum AST levels. Control, control group. Model, model group. AEPE-L, AEPE low-dose group. AEPE-M, AEPE middle-dose group. AEPE-H, AEPE high-dose group. Silymarin, silymarin group. The data were expressed as the mean ± SEM (*n* = 6–10). ^#^*p* < 0.05 and ^##^*p* < 0.01 vs. control.  ^*∗*^*p* < 0.05 and  ^*∗∗*^*p* < 0.01 vs. model.

Morphological changes of liver tissues were observed, as shown in Figure 3(b). The liver tissues in the control group were dark red, smooth, and supple which manifested the liver was normal. After CCl_4_ were applied in model group, the liver tissue was pallid with uneven mottle-like and granular lesions in surface. In the groups treated with different doses of AEPE, the liver surface was smooth and granular lesions were ameliorated, accompanied by soft texture and relatively dark red color.

H&E staining was performed to evaluate the protective effects of AEPE on liver injury by treatment with CCl_4_ in rats. The pathological morphology of liver collected from the different groups was illustrated in Figure 3(c). The hepatic lobules of control rats were intact, and the hepatocytes were arranged trimly without visible abnormalities. However, extensive diffuse necrosis of hepatocytes, steatosis, inflammatory infiltration, sinusoid congestion, and balloon-like changes were detected in hepatic tissue of model rats, which demonstrated the liver fibrosis model was successfully established. Via treatment with AEPE or silymarin, liver tissue injury was significantly repaired with well-observed intact liver tissue structure, homogenized hepatocyte size, slight degeneration, and inflammatory infiltration. AEPE exposure greatly improved liver fibrosis (Table 1). The average severity scores for liver fibrosis in AEPE-treated rats were markedly reduced compared to CCl_4_ controls.

### 3.3. AEPE Improved the Liver Injury in Hepatic Fibrosis Rats

As shown in Figure 3(d), liver index of model rats increased significantly compared to normal control. Meanwhile, compared with the model group, the AEPE medium- and high-dose group can reduce significantly the liver index of model rats, indicating that AEPE can improve liver congestion and swelling caused by CCl_4_.

To investigate the protective effect of AEPE on rat hepatic fibrosis induced by CCl_4_, liver function markers including AST and ALT were detected primarily. As shown in Figure 3(e) and 3(f), the serum levels of ALT and AST in the model group increased significantly, indicating that CCl_4_ successfully induced liver injury in rats. The AEPE can significantly reduce rat serum ALT and AST level in a dose-dependent manner. The results show that AEPE possessed protection of liver cells and improvement of liver function.

### 3.4. Effects of AEPE on Collagen Deposition

**Figure 4:**
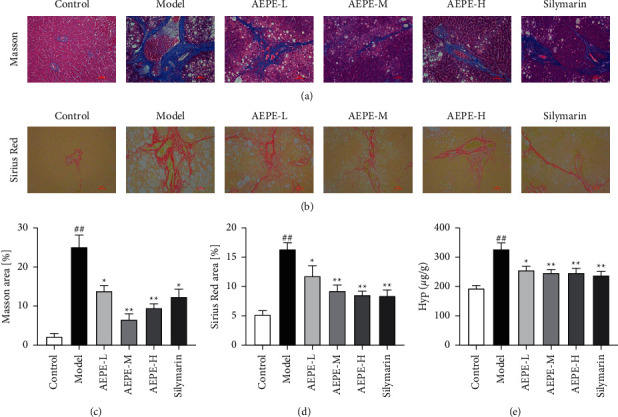
AEPE inhibited CCl_4_-induced rat liver fibrosis and the hydroxyproline content in rats. (a) Representative rat livers stained with Masson (200x). (b) Representative rat livers stained with Sirius Red (200x). (c) The positive regions of Masson staining. (d) The positive regions of Sirius Red staining. (e) Hyp contents of liver tissues. Scale bar = 50 *μ*m. Data were expressed as the mean ± SEM (*n* = 6–10). ^#^*p* < 0.01 and ^##^*p* < 0.01 vs. control;  ^*∗*^*p* < 0.05 and  ^*∗∗*^*p* < 0.01 vs. model.

Hepatic fibrosis was usually accompanied by deposition of collagen components which reflect the degree of fibrosis progression [28]. To detect the distribution of fibrotic tissue, Masson staining and Sirius red staining were applied and relevant results were shown in Figures 4(a)–4(d). There were almost no obviously collagen proliferation in control rat, while plenty of collagens were generated and accumulated in the portal and sinusoidal regions in the model group. AEPEs supplementations notably alleviated the expression of collagen fibers. In particular, 1.8 g/kg AEPE showed significantly better effect in improving these pathological changes. Meanwhile, as shown in Figure 4(e), the Hyp level in the liver tissue of the model animals was significantly increased (*p* < 0.01) compared to the control group. However, the administration of AEPEs and positive control silymarin decreased the upregulated Hyp contents (*p* < 0.05). There are no statistical differences among the three doses of the AEPE groups. These data indicate that AEPEs possessed the function of reducing the expression of collagen fibers in the liver tissue of fibrotic rats.

### 3.5. Effect of PE on LN, HA, IV-C, and PCIII Levels in Serum

Clinically, LN, HA, IV-C, and PCIII were normally applied to evaluate the development of hepatic fibrosis due to the high expression of them in patients with hepatic fibrosis [29]. As shown in Table 2, the expression of LN, HA, IV-C, and PCIII levels was significantly increased in rat serum after being treated by CCl_4_ (*p* < 0.01). Compared with the model group, the level of LN, PCIII, and IV-C in serum was decreased significantly after treatment with 0.9, 1.8, and 3.6 g/kg AEPE (*p* < 0.05), and HA was reduced in the groups treated with 1.8 and 3.6 g/kg of AEPE (*p* < 0.05). The results indicate that AEPE could impede the development of hepatic fibrosis.

### 3.6. Effects of AEPE on Hepatic Stellate Cell Activation

**Figure 5:**
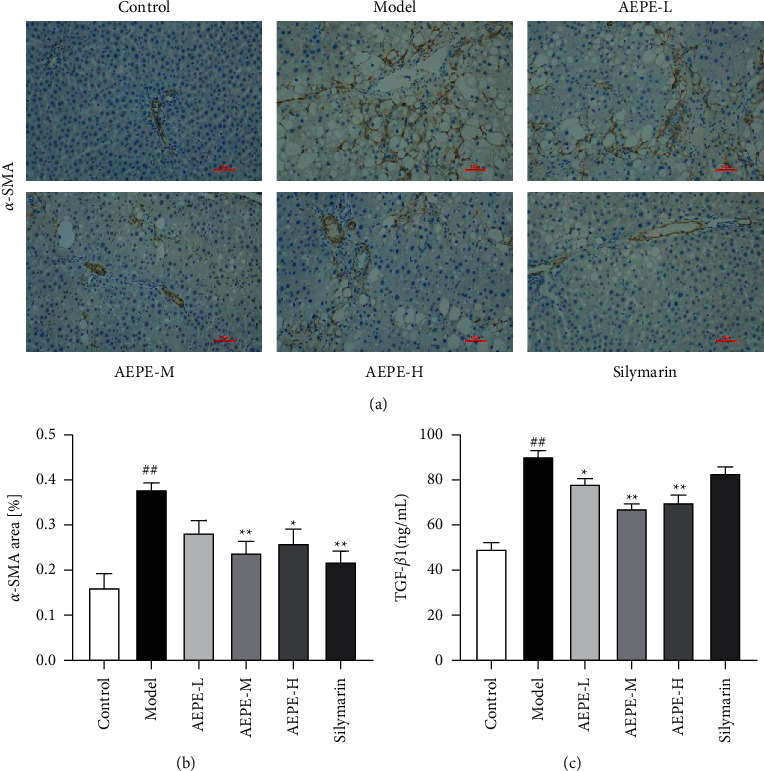
AEPE inhibited HSCs activation in CCl_4_-induced live fibrosis rats. (a) Immunohistochemical analysis of *α*-SMA (200x). (b) *α*-SMA expression positive area. (c) TGF-*β*1 contents of serum. Data were expressed as the mean ± SEM (*n* = 6–10). ^#^*p* < 0.05 and ^##^*p* < 0.01 vs. control;  ^*∗*^*p* < 0.05 and  ^*∗∗*^*p* < 0.01 vs. model.

TGF-*β*1 is a crucial cytokine which could activate HSC to cause liver fibrosis [30]. After induction of CCl_4_, the levels of TGF-*β*1 in the model rats were almost twice that of the control rat (*p* < 0.01). The PE low-, medium-, and high-dose groups can reduce the release of TGF-*β*1, and the best effect is treatment with 1.8 g/kg PE (*p* < 0.01). However, the expression of TGF-*β*1 in silymarin group has no significant difference compared to model group (Figure 5(c)).

*α*-SMA is one of the main marker proteins of HSC activation [29]. Results of the immunohistochemical staining analysis indicated an increased expression of *α*-SMA mainly located in liver parenchymal cells and cytoplasm in liver tissue of the model group compared to the control group. Compared with the model group, the upregulated expression of *α*-SMA in the interstitium of the liver tissue was blocked significantly by the treatment with silymarin and AEPE (0.9, 1.8, and 3.6 g/kg). The results were illustrated in Figure 5.

### 3.7. Effect of AEPE on Liver Oxidative Stress in Hepatic Fibrosis Rats Induced by CCl_4_

**Figure 6:**
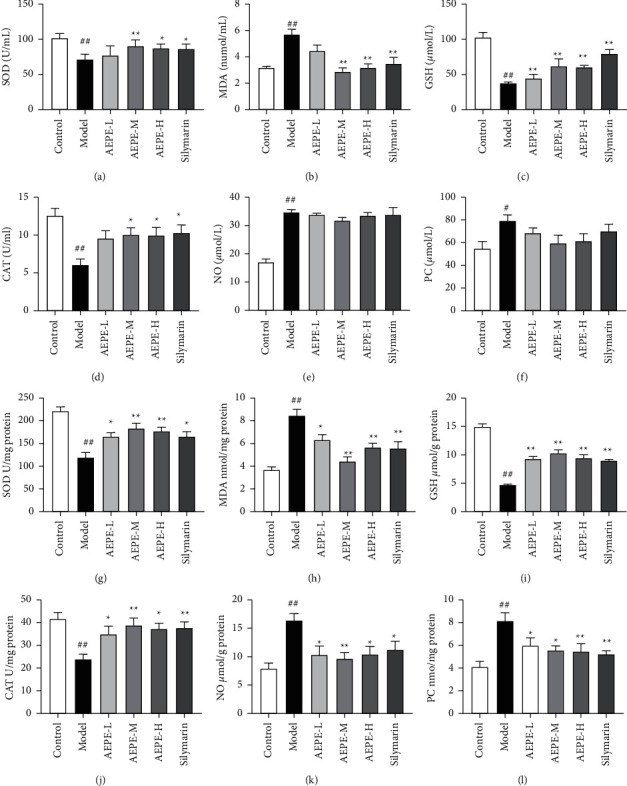
Effects of AEPE on oxidative stress indexes in the serum and liver of rats. The levels of SOD (a), MDA (b), GSH (c), CAT (d), NO (e), and PC (f) in the serum of rats. The levels of SOD (g), MDA (h), GSH (i), CAT (j), NO (k), and PC (l) in the liver tissue of rats. Data were expressed as the mean ± SEM (*n* = 6–10). ^#^*p* < 0.05 and ^##^*p* < 0.01 vs. control; ^*∗*^*p* < 0.05 and  ^*∗∗*^*p* < 0.01 vs. model.

As illustrated in Figure 6, MDA, NO, and PC contents were significantly increased, whereas SOD and CAT activities and GSH levels were remarkably lower in serum and liver tissue in model group than those in control group. Compared with the model group, AEPE-M and AEPE-H reversed the increase of MDA contents and decrease of SOD and CAT activities and GSH levels both in serum and liver tissue. AEPEs and silymarin have no significant influence on NO and PC contents in serum (*p* > 0.05), but they reduced the levels of NO and PC in the liver tissue. In summary, the results indicated that AEPE may protect the liver injury through its dramatically antioxidant activities.

### 3.8. Effect of AEPE on Serum Inflammatory Factor Levels in Rats with Liver Fibrosis

**Figure 7:**
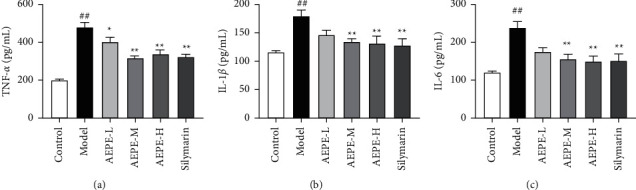
Effects of AEPE on inflammation cytokine secretion in the rat serum of rats. The levels of TNF-*α* (a), IL-1*β* (b), and IL-6 (c) in the serum of rats. Data were expressed as the mean ± SEM (*n* = 6–10). ^#^*p* < 0.05 and ^##^*p* < 0.01 vs. control; ^*∗*^*p* < 0.05 and  ^*∗∗*^*p* < 0.01 vs. model.

Compared to the control group, the results showed that TNF-*α*, IL-1*β,* and IL-6 levels were increased significantly in the CCl_4_-treated group (*p* < 0.01). However, AEPE-treated rats resulted in decreasing in the levels of TNF-*α*, IL-1*β,* and IL-6. These inflammatory factors levels were decreased significantly following treatment with AEPE-M and AEPE-H in silymarin group compared to the model group (*p* < 0.01) (Figure 7).

## 4. Discussion

In the present study, liver fibrosis rat model induced by CCl_4_ was used to investigate the antihepatic fibrosis effects of PE *in vivo*, and the possible mechanism was discovered. CCl_4_ is a typical hepatotoxic drug that leads to liver fatty degeneration and hepatocellular necrosis, which induce liver fibrosis, giving the most classic model to simulate the pathogenesis of human liver fibrosis [31, 32]. In the present experiment, elevated ALT and AST levels indicated liver damage in rats after being injected with CCl_4_ for 8 weeks. H&E staining showed a mass of fatty vacuoles, hepatocellular necrosis, and inflammatory infiltration in liver tissues of model rats. Besides, massive collagen deposition in the model group was observed successfully by Masson and Sirius Red staining. AEPE at various dosages could ameliorate the above zymologic and pathological changes. Hyp is unique amino acid in collagen fibers, which have been regarded as the key biomarkers of fibrosis [33], and AEPEs also decreased the contents of Hyp in liver tissue of model rats. Those results indicate the antihepatic fibrosis activity of PE in vivo.

To further confirm the antiliver fibrosis effect of AEPE, the levels of LN, HA, IV-C, and PCIII in serum were determined. Clinical practice shows that these hepatic fibrotic serum markers are positively related to inflammatory activity and fibrosis of liver tissue [34, 35]. The results have shown that AEPE could significantly decrease the expression of LN, HA, IV-C, and PCIII of CCl_4_-induced rats in a dose-dependent manner. Moreover, excessive expression of *α*-SMA in liver stimulated by CCl_4_ was downregulated after AEPEs treatment. In normal liver tissue, HSCs are in a static state, and HSCs are activated after liver injury [36]. It has been proven that activated HSCs are transformed into myofibroblasts and release ECM, and then excessive ECM deposition and matrix transformation dysfunction eventually lead to liver fibrosis. Spindle-shaped or stellar-shaped myofibroblasts express abundant intracellular proteins [37, 38]. Among them, *α*-SMA is a recognized marker of HSC activation [29]. Accordingly, the immunohistochemistry method is used to detect the expression of *α*-SMA and specify the inhibiting effect of AEPE on the activation of HSCs.

TGF-*β*1 is a crucial target of liver fibrosis, which can activate HSC and regulate the synthesis and degradation of ECM [30, 39]. After stimulation by the cytokine TGF-*β*1, HSCs change from nonproliferative cells to proliferation-activated cells, leading to liver fibrosis [40]. The biological effect of this is mainly realized through the TGF-*β*1/Smad signaling pathway [41]. In this experiment, the TGF-*β*1 content of rats in the AEPE gavage group was significantly lower than that in the CCl_4_ group. Therefore, these results indicated that AEPE can alleviate CCl_4_-induced liver fibrosis by inhibiting the activation of HSCs and possibly through the TGF-*β*1/Smad signaling pathway.

Oxidative stress has been deemed as an important factor of CCl_4_-induced liver fibrosis, which could damage the homeostasis of cellular structure and accelerate the synthesis of collagen via toxicological cascade reaction [4, 24, 42]. In the present study, the HPLC analysis identified 7 major polyphenols ingredients in AEPE (shown in Figure 2.). Among them, gallic acid and ellagic acid have been reckoned as strong antioxidants by increased antioxidant enzyme activities and scavenged free radicals [43, 44]. Thus, we speculated that inhibiting oxidative stress is one of the crucial biological mechanisms of AEPE in antiliver fibrosis. As expected, AEPE decreased hepatic MDA, NO, and PC contents and increased SOD, CAT activities and GSH levels in rats with liver fibrosis. Therefore, rats treated by AEPE may enhance the oxidant defense capacity of liver tissue according to the results.

Finally, in order to explore the role of WEPE in the inflammatory response of liver fibrosis, we evaluated the levels of TNF-*α*, IL-1*β,* and IL-6 in the rat serum. Proinflammatory cytokines participate in inflammation, fibrosis, and cancer development associated with hepatocytes apoptosis and steatosis [44]. In several experimental models of hepatic fibrosis, those proinflammatory mediators are regarded as the major hepatotoxic mediators which mainly contribute to HSC activation and ECM generation [34, 45]. Some reports stated that various extracts from PE cause inhibition of the production of TNF-*α*, IL-1*β,* and IL-6 in atherosclerosis or neuronal cellular model [46, 47]. Overall, the data from the present study have shown that AEPE exerts a protective effect on liver cells by reducing the level of inflammatory factors, which were consistent with the inflammatory cell infiltration found in H&E staining.

As the main secondary metabolite in PE, gallic acid has been confirmed to have antiliver fibrosis activity in vivo [43]. However, the bioactivity of single component in medicinal plants cannot explain its complex pharmacological mechanism. Meanwhile, both previous and present research results have shown that there are massive polysaccharides contained in PE, and it also possesses diversiform biological activities, such as antioxidation, anti-inflammation, and immune regulation. Due to the complexity of the chemical components and pharmacological activities of AEPE, the synergetic antiliver fibrosis mechanism of multiple types of active ingredients in AEPE needs to be further clarified, which will provide scientific basis for the discovery of antiliver fibrosis combination drugs.

## 5. Conclusion

The present study confirmed the antihepatic fibrosis effect of AEPE in vivo and proved that AEPE can reduce CCl_4_-induced liver fibrosis and improve liver function by reducing ECM accumulation, HCS activation, oxidative stress, and inflammatory response. These results provide experimental basis for further research on the molecular mechanism of AEPE against liver fibrosis. Taken together, as both medicinal and edible plants, PE can be used in nutraceuticals and pharmaceutical applications to prevent chronic liver disease from further evolving into liver fibrosis.

## Figures and Tables

**Table 1:** AEPE effect on histopathological of CCl_4_-induced liver fibrosis in rats.

Group	Severity score of hepatic fibrosis					Average
	0	1	2	3	4	
Control	6	0	0	0	0	0.00 ± 0.00
Model	0	0	0	2	4	3.67 ± 0.52^##^
AEPE-L	0	0	3	2	1	2.67 ± 0.82^*∗∗*^
AEPE-M	0	1	2	3	0	2.33 ± 0.82^*∗∗*^
AEPE-H	0	0	3	3	0	2.50 ± 0.55^*∗∗*^
Silymarin	0	1	2	2	1	2.50 ± 1.05^*∗∗*^

Data were expressed as the mean ± SEM (*n* = 6). ^##^*p* < 0.01 vs. Control;  ^*∗*^*p* < 0.05, and  ^*∗∗*^*p* < 0.01 vs. model.

**Table 2:** AEPE ameliorates CCl_4_-induced liver fibrosis in rats serum LN, HA, IV-C, and PCIII levels, respectively.

Groups	LN (ng/mL)	HA (ng/mL)	IV-C (ng/mL)	PCIII (ng/mL)
Control	104.38 ± 4.73	82.72 ± 9.44	5.48 ± 0.35	9.57 ± 1.09
Model	156.53 ± 3.51^##^	103.64 ± 7.35^##^	7.09 ± 0.93^##^	12.97 ± 1.44^##^
AEPE-L	129.7 ± 11.76^*∗∗*^	94.73 ± 8.30	5.79 ± 0.38^*∗∗*^	11.32 ± 0.57^*∗*^
AEPE-M	134.72 ± 7.04^*∗∗*^	90.24 ± 5.37^*∗*^	5.69 ± 0.39^*∗∗*^	11.21 ± 0.64^*∗*^
AEPE-H	126.8 ± 10.76^*∗∗*^	90.49 ± 6.21^*∗*^	5.70 ± 0.40^*∗∗*^	11.31 ± 0.66^*∗*^
Silymarin	134.38 ± 7.93^*∗∗*^	83.41 ± 6.97^*∗∗*^	5.89 ± 0.40^*∗∗*^	12.06 ± 1.20

Data were expressed as the mean ± SEM (*n* = 6–10). ^##^*p* < 0.01 vs. control;  ^*∗*^*p* < 0.05,  ^*∗∗*^*p* < 0.01 vs. model.

## Data Availability

Datasets of the study are available from the corresponding author on reasonable request.
