# Corrigendum to “Hepatoprotective Effect and Potential Mechanism of Aqueous Extract from *Phyllanthus emblica* on Carbon-Tetrachloride-Induced Liver Fibrosis in Rats”

**DOI:** 10.1155/ecam/9753651

**Published:** 2025-08-06

K. Yin, X. Li, X. Luo, Y. Sha, P. Gong, J. Gu, and R. Tan, “Hepatoprotective Effect and Potential Mechanism of Aqueous Extract from *Phyllanthus emblica* on Carbon-Tetrachloride-Induced Liver Fibrosis in Rats,” *Evidence-Based Complementary and Alternative Medicine* 2021 (2021): 5345821, https://doi.org/10.1155/2021/5345821.

In the article titled “Hepatoprotective Effect and Potential Mechanism of Aqueous Extract from *Phyllanthus emblica* on Carbon-Tetrachloride-Induced Liver Fibrosis in Rats,” there were errors in Figures 1, 2.

Figure 1 should show the images of the chemical structures of the 7 chemical components in AEPE. The corrected figure is shown below:

Figure 2 should show the HPLC chromatograms of the AEPE sample and the 7 standard compounds. The corrected figure is shown below:

The authors confirm that these errors do not affect the results and conclusion of the article.

We apologize for these errors.

## Figures and Tables

**Figure 1:**
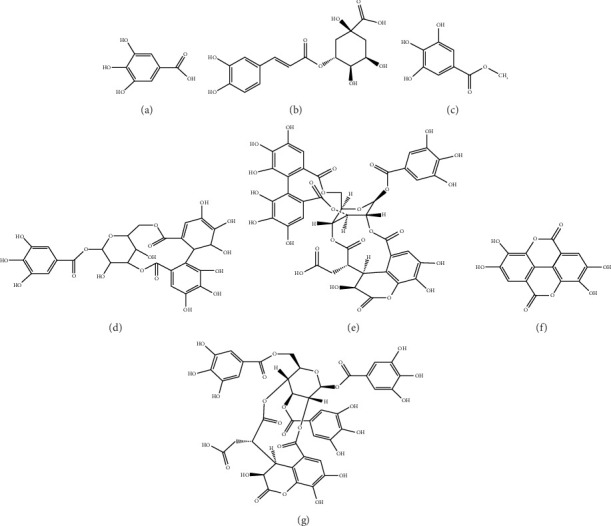
Structural formulas for qualitative detection of 7 chemical components in AEPE. (a) Gallic acid; (b) chlorogenic acid; (c) methyl gallate; (d) corilagin; (e) chebulagic acid; (f) ellagic acid; (g) chebulinic acid.

**Figure 2:**
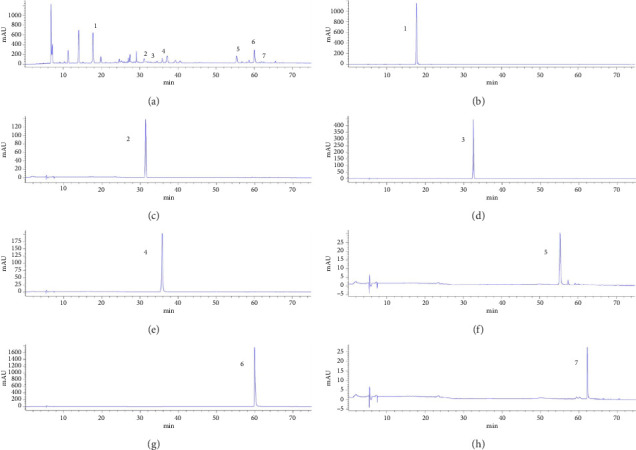
Representative high-performance liquid chromatograms of AEPE sample (a) and standard compounds (b), (c), (d), (e), (f), (g), (h). (1) Gallic acid; (2) chlorogenic acid; (3) methyl gallate; (4) corilagin; (5) chebulagic acid; (6) ellagic acid; and (7) chebulinic acid.

